# Adaptive reinforcement learning for task scheduling in aircraft maintenance

**DOI:** 10.1038/s41598-023-41169-3

**Published:** 2023-10-03

**Authors:** Catarina Silva, Pedro Andrade, Bernardete Ribeiro, Bruno F. Santos

**Affiliations:** 1 CISUC-Centre Informatics and Systems, Informatics Engineering Department, University of Coimbra, Polo II, Coimbra, 3004-531 Coimbra, Portugal; 2 Air Transport and Operations, Faculty of Aerospace Engineering, Delft University of Technology, Delft, The Netherlands

**Keywords:** Engineering, Aerospace engineering

## Abstract

This paper proposes using reinforcement learning (RL) to schedule maintenance tasks, which can significantly reduce direct operating costs for airlines. The approach consists of a static algorithm for long-term scheduling and an adaptive algorithm for rescheduling based on new maintenance information. To assess the performance of both approaches, three key performance indicators (KPIs) are defined: Ground Time, representing the hours an aircraft spends on the ground; Time Slack, measuring the proximity of tasks to their due dates; and Change Score, quantifying the similarity level between initial and adapted maintenance plans when new information surfaces. The results demonstrate the efficacy of RL in producing efficient maintenance plans, with the algorithms complementing each other to form a solid foundation for routine tasks and real-time responsiveness to new information. While the static algorithm performs slightly better in terms of Ground Time and Time Slack, the adaptive algorithm excels overwhelmingly in terms of Change Score, offering greater flexibility in handling new maintenance information. The proposed RL-based approach can improve the efficiency of aircraft maintenance and has the potential for further research in this area.

## Introduction

In aviation, maintenance is typically carried out preventively by scheduling periodic maintenance checks for each aircraft. These checks encompass a pre-established set of routine tasks, as well as non-routine work that may vary based on maintenance needs. Aircraft have thousands of components and systems that need to be maintained at certain intervals that can be determined by the number of hours of flight (FH), the number of round trips or cycles (FC), and the number of days (DY). However, most tasks have a periodicity that does not match the maintenance checks. In these cases, they are either scheduled much earlier than the end of their interval, increasing the maintenance costs in the long term, or they are scheduled in smaller maintenance slots, which may considerably increase the ground time. According to^1^, maintenance expenses account for roughly 10% of an airline’s total operating costs. Additionally, a relevant part of the maintenance scheduling work is done with a manual approach, which can be extremely time-consuming and lead to sub-optimized maintenance plans. Therefore, it is important to improve current maintenance practices. Aircraft maintenance has been a research focus over the years, in which most studies focus on two goals: cost reduction and increased aircraft availability. An innovative formulation for aircraft re-assignment and maintenance scheduling was proposed in^2^ that uses a heuristic solution capable of reducing maintenance costs. The daily scheduling of maintenance tasks was the focus in^3^. The goal is to optimize the selection of tasks to be performed every day on a fleet of fighter aircraft to maximize their availability for future missions. In^4^, the authors opt for the traditional block packaging method followed by many airlines and use a clustering algorithm, grouping multiple tasks together based on their similarities. A constructive heuristic is used in^5^ to solve the problem of scheduling maintenance tasks into checks, formulated as a time-constrained variable-sized bin packing problem. reinforcement learning (RL) has also been used for maintenance optimization in multiple domains. In previous research, a Q-learning algorithm was suggested in^6^ to determine whether a maintenance job should be executed at each step. Additionally, RL was applied to improve maintenance scheduling in a flow line system, as proposed in^7^. In the aviation domain, Hu et al.^8^ utilizes RL to enhance long-term maintenance decisions for aircraft, while in^9^ a Deep Q-learning algorithm to maximize aircraft utilization by optimizing the scheduling policy for A and C-checks is proposed. On the same line, in^10^ a deep reinforcement learning for predictive aircraft maintenance using probabilistic Remaining-Useful-Life prognostics is presented, by estimating the distribution of remaining useful life using Convolutional Neural Networks with Monte Carlo dropout, updating the prognostics over time, as more measurements become available, and setting the maintenance planning problem as a Deep Reinforcement Learning problem where maintenance actions are triggered based on the estimates of the RUL distribution.

This paper proposes two reinforcement learning (RL) algorithms aimed at optimizing maintenance task scheduling. The first algorithm is a static scheduling approach, which prioritizes the long-term scheduling of routine tasks into maintenance opportunities. Instead of scheduling pre-packaged sets of tasks, this algorithm schedules individual tasks into maintenance checks and smaller maintenance slots to minimize ground time and maximize component utilization. To minimize access costs, tasks with similar access requirements are grouped together in the same check. The second algorithm is an adaptive scheduling approach that modifies short-term plans when new maintenance data is available, such as newly identified faults and remaining useful life (RUL) prognostics. This algorithm builds upon the current maintenance plan, only making the necessary modifications instead of creating an entirely new plan each time, which is impractical in real-world scenarios. Three scenarios—mild, medium, and aggressive—were devised to compare both algorithms under varying levels of maintenance load. The scenarios differ in the number and severity of new maintenance events considered.

Three key performance indicators (KPIs) are proposed to evaluate the performance of both approaches: Ground Time, which denotes the amount of hours aircraft remain on the ground; Time Slack, measuring the proximity of tasks to their due dates; and Change Score, quantifying the similarity between the initial and adapted maintenance plans when new information emerges. All three key performance indicators (KPIs) are aimed at minimization.

The results demonstrate the efficiency of RL in producing effective maintenance plans, with the algorithms working in synergy to establish a strong basis for routine tasks and real-time responsiveness to emerging information. While the adaptive algorithm falls slightly behind the static algorithm in terms of Ground Time and Time Slack, both critical Key Performance Indicators (KPIs) in aircraft maintenance, it excels significantly in the Change Score metric. This metric gains paramount importance in current maintenance scenarios, as it measures the impact of plan modifications on various aspects such as maintenance personnel, equipment/shop, space, and parallel capacity^11–13^. These findings underscore the adaptive algorithm’s exceptional adaptability to handle unexpected changes, making it highly valuable in dynamic maintenance environments where flexibility is of utmost importance.

The organization of this paper is as follows: “Reinforcement learning” provides background information on RL. “Static scheduling algorithm” and “Adaptive scheduling algorithm” present the static scheduling algorithm and the adaptive scheduling algorithm, respectively. “Maintenance scenarios simulation” explains the process of simulating maintenance scenarios. “Results” discusses the results obtained in this study. Finally, “Conclusions and future work” summarizes the work completed and suggests future areas for exploration.

## Reinforcement learning

Reinforcement learning (RL) is a subfield of Machine Learning that involves an agent interacting with an environment by making a series of decisions in pursuit of a specific objective^14^. The agent receives a reward based on the quality of each action taken after making a decision. The goal is for the agent to learn from its decisions and continuously choose actions that result in higher rewards. The RL agent begins in an initial state and continues to make decisions until it reaches a terminal state. This sequence is called an episode, and the agent’s training is completed through multiple episodes.

Reinforcement learning (RL) is a formal framework in machine learning where an agent interacts with an environment, taking actions to reach a specific goal^14^. RL is represented as a Markov Decision Process (MDP), defined as a tuple (*S*, *A*, *P*, *R*, $\gamma$), where *S* denotes the set of states, *A* is the action set, *P* is the transition function, *R* is the reward function, and $\gamma$ is a discount factor, which gives less importance to future rewards. At each time step *t*, the agent is in a state $s_t$ and selects an action $a_t$. The agent then moves to the next state $s_{t+1}$ and receives a reward $r_t$, indicating the quality of the action chosen. The agent follows a policy, mapping from states to actions, to select an action, and the policy defines the agent’s behavior.

### Q-learning

Q-Learning^15^ is a widely used RL algorithm that utilizes a Q-table, a look-up table with a shape of [states, actions]. Each entry in the Q-table represents the Q-value *Q*(*s*, *a*), which indicates the quality of selecting an action *a* in a state *s*. Assuming that the agent follows a greedy policy, it selects the action with the highest Q-value in the current state. After each action, the reward received is used to update the corresponding Q-value in the Q-table using the following update function, where $\alpha$ is the learning rate (Eq. 1):

1 $$\begin{aligned} Q(\!s_t,\!a_t\!)\!=\!Q(\!s_t,\!a_t\!)+\alpha \!\left[ \!r_{t+\!1}\!\! +\!\gamma \max _{a}Q(\!s_{t+\!1},\!a)\!-\!Q(\!s_t,\!a_t\!)\!\right] \!. \end{aligned}$$

### Deep Q-learning

The main drawback of the traditional Q-table algorithm is that it does not scale with larger and more complex problems. The go-to solution for these types of problems is the variant of Deep Q-learning, which uses Artificial Neural Networks to obtain approximations for the Q-values. Deep Q-learning uses the concept of experience replay to train the agent. After each decision, the corresponding experience is saved in a buffer, usually called replay memory. An experience is a tuple ($s_t$, $a_t$, $r_t$, $s_{t+1}$) containing the current state, $s_t$, the action selected, $a_t$, the reward received, $r_t$, and next state, $s_{t+1}$. During training, a random batch of experiences is sampled from the replay memory and utilized to update the neural network. This process, known as experience replay, addresses two common issues associated with Deep Q-learning. Firstly, the agent tends to forget past experiences as time passes, and secondly, consecutive experiences are often correlated. Experience replay helps to mitigate both these problems.

RL is usually described as a trial-and-error approach in which an agent learns from his own decisions. Ideally, the agent should collect a wide variety of experiences and properly use the entire state-action space. In most cases, choosing the best action every time is not the best approach, as it can often lead to suboptimal solutions. In RL, there is a trade-off between exploration and exploitation. Exploration involves choosing a random action to gather new information, while exploitation involves selecting the best-known action based on past experience. To balance exploration and exploitation, this study employs the $\epsilon$-greedy strategy, where the agent selects the best action in the current state with probability $1 - \epsilon$ and a random action with probability $\epsilon$. At the start of training, $\epsilon$ is set to 1 to encourage exploration, and its value decreases over time, favoring exploitation. This ensures that the agent explores the environment sufficiently while learning to optimize its actions based on past experiences.

The Double Q-learning method introduced in^16^ was also used in this work. As can be gleaned from Algorithm 1^17^, a second neural network called the target network is added to remove over-estimations known to occur in the action values^17^.
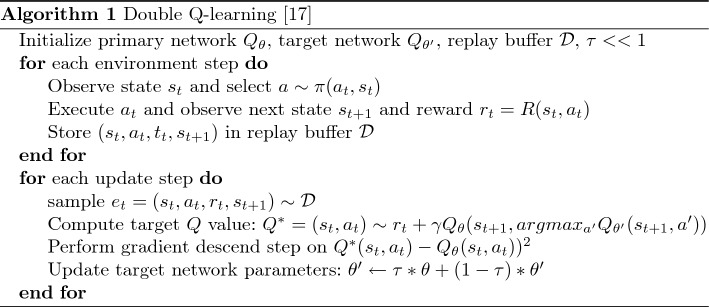


To estimate the action values, the target network is employed, while the online network is responsible for selecting the action. As the weights of only the online network are being updated during training, the target network’s weights are periodically synchronized with those of the online network. This allows for more stable training, as it prevents the target network’s values from changing too rapidly.

## Static scheduling algorithm

Maintenance routine tasks are usually clustered together in work packages, and performed maintenance checks^18^. The tasks clustered in these work packages represent a significant portion of the maintenance plan. Therefore, optimizing the scheduling of these tasks is essential to obtaining a good plan, and that is the goal of the static scheduling algorithm. The RL agent aims to reduce the fleet ground time and time slack. Ground time is the number of hours an aircraft stays grounded performing maintenance. Time slack can be defined as the time difference between the due date of the task and its scheduled day. Achieving low time slacks reduces the frequency in which tasks must be performed, which in the long term contributes to a higher aircraft availability for operations and lower maintenance costs.

The static scheduling algorithm takes as input the maintenance routine tasks, the flight plan, the check schedule, and the average fleet utilization to generate a maintenance plan. The check schedule is obtained using the work developed in^9^, which schedules A- and C-checks for the fleet. Although intervals are aircraft type-specific, typically, A-checks occur every 1500 FH or three months and usually last 24 h, and C-checks occur every 18,000 FH or 3 years and can last several weeks. The long intervals between checks, which are matched by the majority of routine tasks, make this algorithm more appropriate for long-term maintenance scheduling. In an attempt to have a closer representation of a real maintenance scenario, the following assumptions are considered in this approach:

**A1.**: Aircraft utilization is constant and measured in FH and FC.
**A2.**: Maintenance can only be performed at the base airport.
**A3.**: Maintenance is always performed at a hangar.
**A4.**: Maintenance slots for an aircraft require it to be on the ground for at least 4 consecutive hours.
**A5.**: A maintenance slot cannot start or end 30 min away from a flight due to logistics reasons.

**Figure 1:**
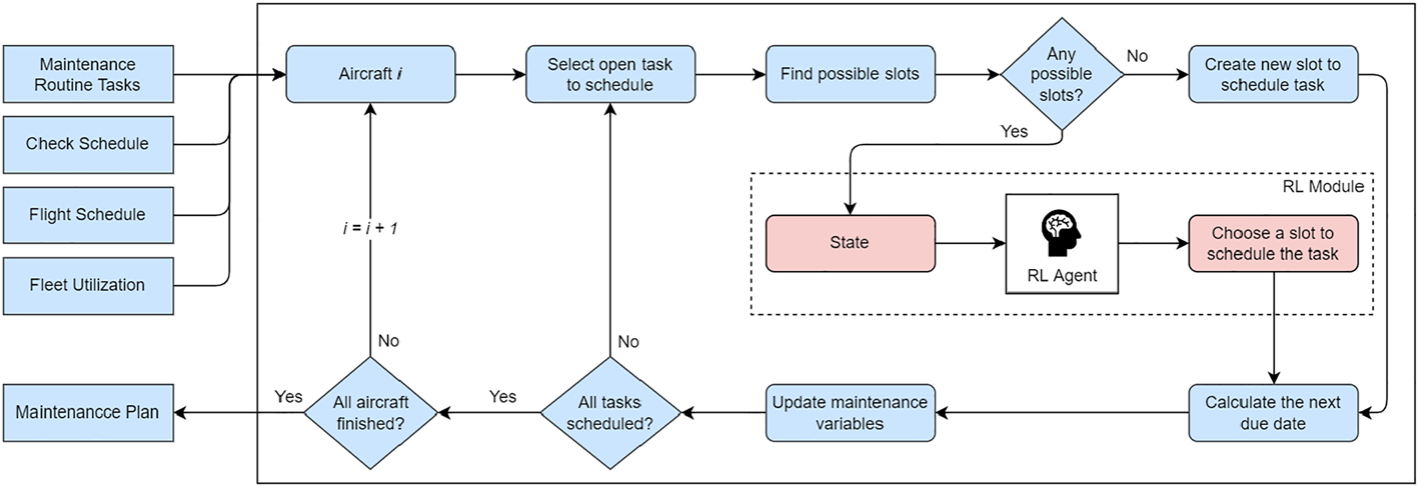
Static scheduling algorithm overview.

Figure 1 shows an overview of the static scheduling algorithm, which works as follows: an open task is selected to be scheduled at each step. A task is considered open if its next due date is within the defined horizon. Then, all possible slots to schedule the task are retrieved. These correspond to the slots scheduled before the task due date that have enough time left to include the task. A new one is created if there are no slots to schedule the task before it goes due. Otherwise, the RL agent allocates the task to an existing slot. Finally, the plan and maintenance variables are updated. The next due date for the current task is computed based on the date the task is scheduled to be performed and the corresponding maintenance interval of the task. An aircraft maintenance schedule is finished when all its tasks are scheduled for the entire horizon. This process is repeated for every aircraft in the fleet.

A new slot is created if there are no other slots before the task due date or if those slots are already full. The chosen period for the slot corresponds to the closest period available before the task due date. In addition to the flight plan, two constraints influence this period selection:

- There is a maximum number of grounded aircraft at the same time to ensure operation demands are always satisfied. A new slot cannot be scheduled for periods that have reached this limit.
- There is a minimum number of days between two consecutive groundings for the same aircraft to balance the fleet maintenance requirements and avoid cases where the same aircraft is grounded multiple times in the same week.

It is also important to note that tasks are only packaged into smaller slots if no checks are available. The idea is to reduce the number of groundings by doing most of the work at A and C-checks.

**Figure 2:**
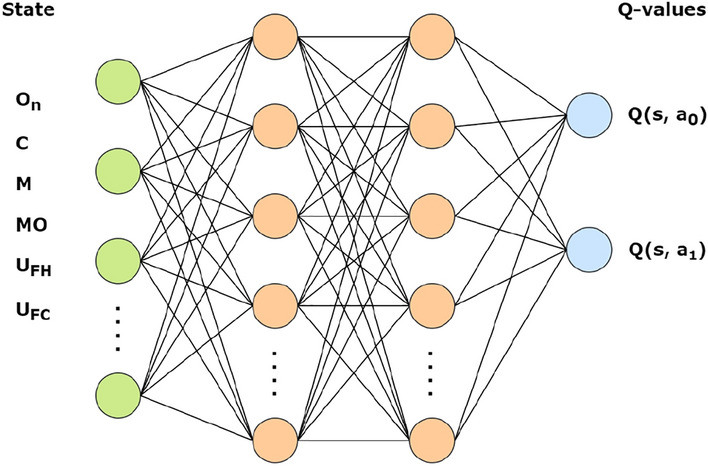
Representation of the static algorithm neural network architecture.

Figure 2 shows a representation of the neural network. The role of the RL agent is to choose the slot to assign to each task. It needs to have enough information regarding the packaged task and candidate slots. Therefore, the current open task ($O_n$), the possible A/C-checks (*C*) and smaller slots (*M*) to schedule the task, the maintenance opportunities (*MO*), and the aircraft utilization in FH and FC ($U_{FH}$, and $U_{FC}$) are all part of the state. Maintenance opportunities can be defined as the possible periods when a slot could be created that meets the constraints mentioned previously.

The agent in this task can perform two actions. The first action involves assigning the task to the slot that is closest to its due date, thereby reducing the time slack. The second action involves selecting the slot that has the highest number of tasks already assigned with the same panel requirements. This action reduces the access cost of that particular maintenance visit. These two actions combined actually concur to achieve the goal of the optimization.

The reward function determines the reward that the agent receives after deciding on scheduling a task, *n*, in a slot, *m*, and is defined in Eq. (2):

2 $$\begin{aligned} R = {\left\{ \begin{array}{ll} \dfrac{Sh_n}{Th_m} - \%I \times d, &{} \text {if task requires access} \\ - \%I \times d, &{} \text {otherwise } \end{array}\right. } \end{aligned}$$

The first case of the reward function is used if the task, *n*, being scheduled, requires at least one access panel to be opened. It contains a positive term consisting of the percentage of shared access hours with other tasks in the selected slot, obtained by dividing task *n* shared access hours, $Sh_n$, by the total access hours in that slot, $Th_m$. These hours correspond to the opening and closing times of the respective panels. The goal of minimizing time slack is represented in the second term, which gives a negative signal equal to the percentage of interval unused multiplied by the task duration. This duration is included because higher-duration tasks usually have higher costs. These tasks should have a larger contribution to the reward because a higher time slack means they must be performed more often in the long term. The second case is for tasks without access requirements, as it only contains the term regarding the time slack goal.

## Adaptive scheduling algorithm

Maintaining an efficient maintenance plan in the face of a constant influx of new information is a significant challenge for maintenance planners. The adaptive scheduling algorithm is designed to address this challenge by dynamically updating the maintenance plan as new information becomes available, such as new faults and RUL prognostics. The ultimate aim is to ensure that the maintenance plan remains as efficient as possible. Contrary to the static algorithm, the idea is to use the current plan as a baseline and only perform the necessary changes. In addition to the current maintenance plan, the adaptive algorithm also takes as input the flight plan for the fleet and the open tasks to be scheduled, created by the simulation of new faults and RUL prognostics. This approach follows the same assumptions defined in the static scheduling algorithm along with three new ones (**A6**–**A8**) relating to the newly simulated maintenance events:

**A1.**: Aircraft utilization is constant and measured in FH and FC.
**A2.**: Maintenance can only be performed at the base airport.
**A3.**: Maintenance is always performed at a hangar.
**A4.**: Maintenance slots for an aircraft require it to be on the ground for at least 4 consecutive hours.
**A5.**: A maintenance slot cannot start or end 30 min away from a flight due to logistics reasons.
**A6.**: Faults have a higher priority and must be scheduled as soon as possible.
**A7.**: Tasks with an RUL have the lowest priority and are the last tasks to be scheduled.
**A8.**: Tasks with RUL have flexible due dates, meaning they can be scheduled beyond that date if no other slot is available.

**Figure 3:**
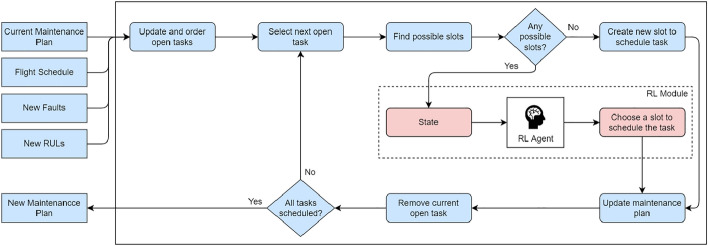
Representation of the adaptive algorithm neural network architecture.

Figure 3 illustrates the adaptive scheduling algorithm. The first step is to update the open tasks based on the new faults and RUL prognostics and order them to define the scheduling priority. Maintenance tasks created to rectify faults have a higher priority and should be scheduled as soon as possible. These tasks are arranged based on the respective fault urgency, with higher priority faults having a higher priority. The remaining tasks are scheduled after and are arranged based on their due date. In the scheduling phase, the RL agent chooses a slot to schedule the task and updates the maintenance plan accordingly. Again, a new task is created when a task cannot be scheduled in any existing slot. This process is repeated for all open tasks.

**Figure 4:**
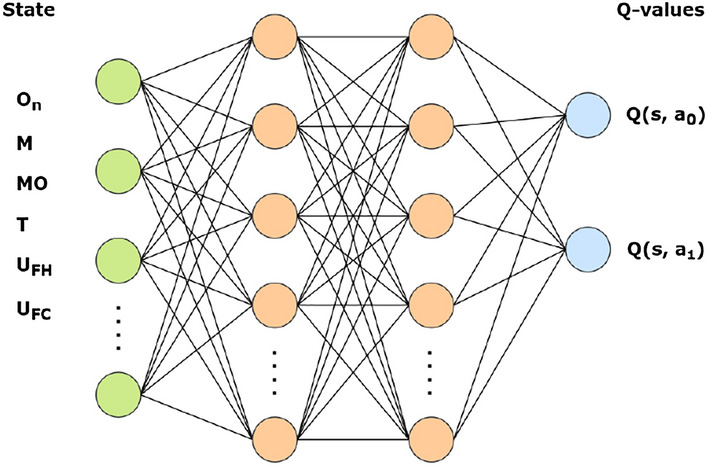
Representation of the adaptive algorithm neural network architecture.

Figure 4 shows a neural network representation. The RL agent should be given enough information to make good decisions. Therefore, the state contains variables such as the current open task ($O_n$), available slots (*M*), possible maintenance opportunities (*MO*), time slack for each available slot (*T*), and the daily aircraft utilization in FH and FC ($U_{FH}$, and $U_{FC}$).

When deciding which maintenance slot to allocate a task, the RL agent can choose between two actions. The first action is to select the slot that minimizes the time slack, i.e., the one closest to the task’s due date. The second action takes into account both the task duration and access cost, with the agent selecting the slot that minimizes the overall aircraft ground time. In cases where a new slot must be created to perform a specific task, the ground time is greatly increased due to the additional time related to logistics.

When the agent performs an action, it receives a reward signal that evaluates the quality of that decision. This reward is a crucial component in the learning process and is typically defined by a reward function:

3 $$\begin{aligned} R = -u-(d+c) \end{aligned}$$

The factor *u* corresponds to the time slack, and the factor $(d + c)$ corresponds to the sum of the task duration, *d*, with any additional cost, *c*. If a task requires other previous tasks, such as opening access panels to reach the component, that extra work falls under the additional cost. Both are negative factors because the goal is to minimize them.

## Maintenance scenarios simulation

The adaptive scheduling algorithm requires new faults and RUL prognostics for training and testing. It aims to plan maintenance for the short term, and in this study, the planning horizon was set to 1 month. Because these new maintenance events are commonly unpredictable and unexpected and, thus, were not available in advance, they were simulated based on past occurrences. The historical data contains crucial metrics such as the weekly average of faults and their urgency level, which serve as a basis to determine the simulation parameters. Therefore, for every week of the maintenance plan, a number of new maintenance events are simulated for each aircraft. A new task is immediately created in response to a fault, and its due date depends on the fault urgency level. The operators’ Master Minimum Equipment List (MMEL) defines four categories of rectification intervals that directly correlate with the urgency level of a fault^19^. These categories are as follows:

- Category A: No standard interval is specified. However, the operator’s approved MMEL specifies the time interval within which items in this category should be repaired, as stated in the “Remarks and Exceptions” column.
- Category B: An urgent rectification is required for items falling under this category, and the repair should be completed within 3 calendar days (excluding the day of discovery).
- Category C: Specifies that items’ rectification should be completed within 10 calendar days (excluding the day of discovery).
- Category D: This category specifies that items should be rectified within 120 calendar days, excluding the day of discovery.

Although Category A does not specify a rectification interval, it represents the highest urgency level. Thus, for the purposes of this simulation, that interval is set to 1 calendar day. For instance, a simulated fault from Category A leads to a new task with a due date set to 1 day after the discovery. Regarding new RUL prognostics, these affect already existing tasks, resulting in updating their due date.

To evaluate the algorithm’s performance under different maintenance loads, three maintenance scenarios were created: mild, medium, and aggressive. The simulation parameters used for each scenario are presented in Table 1.

**Table 1:** Simulation parameters for each maintenance scenario.

	Mild	Medium	Aggressive
New RULs	0	2	4
New faults	3	6	10
A-type faults	0.05	0.05	0.05
B-type faults	0.15	0.15	0.15
C-type faults	0.15	0.15	0.15
D-type faults	0.65	0.65	0.65

Each scenario’s number of new simulated events varies, affecting the fleet maintenance demand. The values correspond to the weekly amount for each aircraft. For example, in a mild scenario, each aircraft has three new faults every week. These faults are discovered on a random day of the week and assume one of the previously mentioned fault categories with a certain probability. The probability of a fault belonging to a certain category is obtained from past data, where lower urgency faults were present in much higher numbers.

The work developed in this paper uses maintenance and operation data from a 16-aircraft Boeing 787 fleet from a major European airline. The maintenance data contains the list of routine tasks for the B787 family, including important details such as their interval, estimated duration, required skills, and required access panels. There is also information about past slots and tasks executed in each of them for 2 years, making it possible to determine the percentage of routine versus non-routine work and the average maintenance duration.

Regarding the operation data, the records of historical aircraft usage are used to calculate the average daily usage for each aircraft, which is essential in calculating the due date of maintenance tasks. The operation data also includes past flights from a 6-month period. Several parameters can be obtained from this flight history and used to simulate a flight plan for the required time horizon, including the weekly number of flights per aircraft, the average flight duration, and the minimum time between consecutive flights. With this data, a flight plan for a two-week period is simulated and repeated over the entire horizon. For the purpose of this work, aircraft are only allowed to perform maintenance at the base airport. Therefore, flights are grouped in routes that begin and end at the base airport, so maintenance can only be performed between these routes.

## Results

This section presents the results obtained with both algorithms regarding the quality of the obtained maintenance plans and the performance of the RL agent. Both algorithms were tested in the three simulated scenarios, and the updated maintenance plan is evaluated with three Key Performance Indicators (KPIs):

- Ground time: amount of hours the fleet spends on the ground performing maintenance during the entire time horizon.
- Time slack: sum of the differences (in hours) between the task due date and its scheduled day for all scheduled tasks in the plan.
- Change score: value between 0 and 1 representing the similarity level between the initial maintenance plan and the new one. A low change score indicates very few changes were made to the initial plan.

The change score is obtained using the percentage of slots and tasks that were changed in the new maintenance plan or, in other words, were scheduled at a different time. Equation (4) represents this calculation.

4 $$\begin{aligned} CS = \dfrac{1}{2}\times \text {\textit{Pct. slots changed}}+ \dfrac{1}{2}\times \text {\textit{Pct. tasks changed}} \end{aligned}$$

Ideally, the change score should be as low as possible when scheduling for the short term. Otherwise, workforce availability, the readiness of parts and tools required for maintenance, and other logistics elements might become a problem due to the lack of time to prepare and react to planning changes.

Table 2 presents the results obtained with the scheduling algorithms regarding the three defined KPIs. Both algorithms received as input the same tasks to schedule in each scenario and produced a 1-month horizon plan. In addition to the three maintenance scenarios mentioned previously, a stationary scenario is also considered, representing the baseline plan obtained without any simulation of new faults or RUL prognostics.

**Table 2:** Results for the scheduling algorithms regarding the predefined KPIs.

KPI	Scheduling algorithm	Maintenance load			
		Stationary	Mild	Medium	Aggressive
Ground time (h)	Static	502	529	601	629
	Adaptive	–	543	627	681
Time slack (h)	Static	234	256	244	230
	Adaptive	–	279	268	255
Change score [0, 1]	Static	–	0.53	0.59	0.70
	Adaptive	–	0.08	0.12	0.18

We see that ground time increases with higher maintenance loads, which is to be expected because, with more maintenance requirements, aircraft will inevitably spend more time on the ground performing maintenance. On the contrary, the time slack decreases with higher maintenance loads. This can be explained by the fact that tasks are more likely to be scheduled closer to their due date with an increased number of scheduled slots. In both of these KPIs, the static algorithm performs better than the adaptive algorithm due to the freedom to create an entirely new plan. However, the change scores indicate that the adaptive algorithm is the better option, especially if the aim is short-term scheduling, where massive changes are not always feasible.

**Figure 5:**
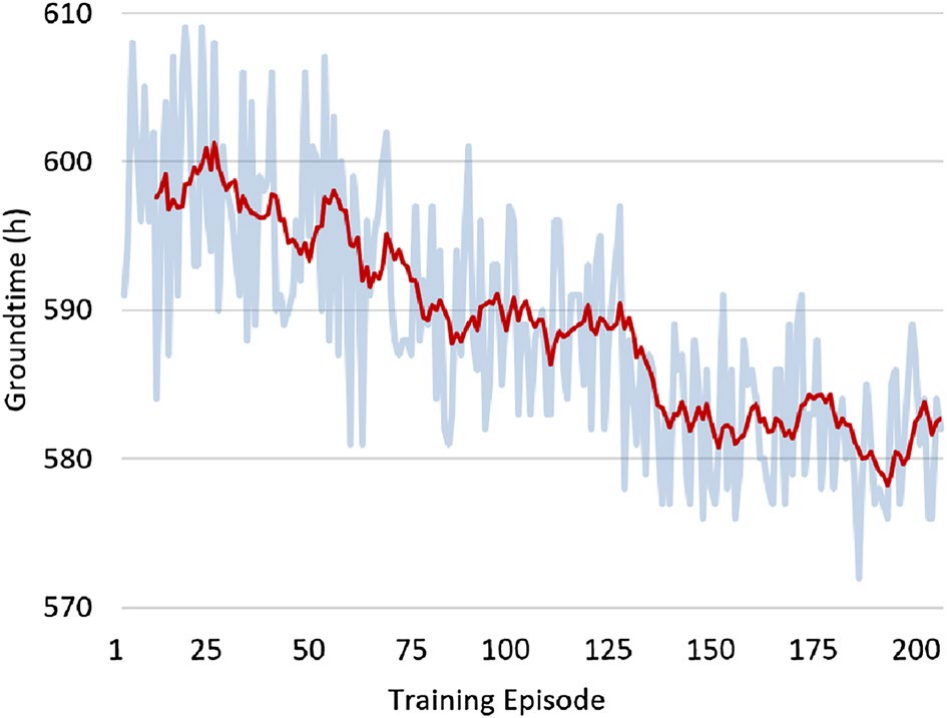
Ground time evolution throughout training.

**Figure 6:**
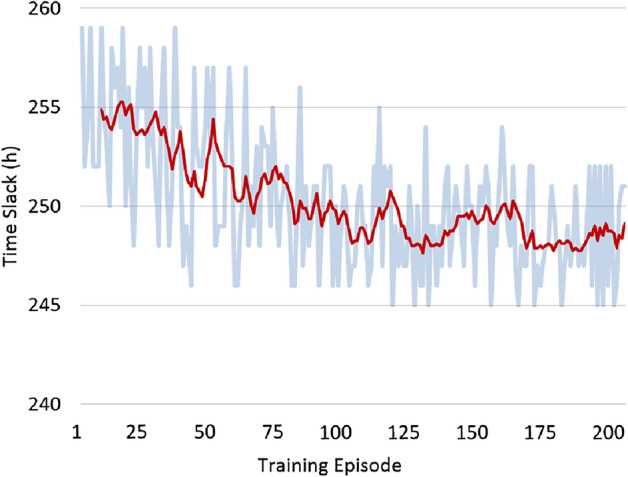
Time slack evolution throughout training.

The development of ground time and time slack during RL agent training can be observed in Figs. 5 and 6, respectively. Due to the limited experience and high exploration levels at the beginning of the training, the quality of the plans initially suffers. However, as the training progresses, the agent gains knowledge and makes better decisions, resulting in improved plan quality.

## Conclusions and future work

This paper proposes two reinforcement learning-based solutions for optimizing the scheduling of maintenance tasks for an aircraft fleet. The first is a static scheduling algorithm for long-term planning, which creates a new maintenance plan for the fleet. The second is an adaptive scheduling algorithm for short-term planning, which optimizes maintenance decisions in response to unexpected events with minor adaptations to the current maintenance plan. The goal is to minimize overall time slack by scheduling tasks as close as possible to their due date and minimizing fleet ground time. Both algorithms utilize Deep Q-learning to train an agent responsible for selecting when to schedule each task.

A dataset with maintenance and operation data from a 16 aircraft fleet was used for the training and testing of the RL agent. Both algorithms were evaluated in several scenarios representing different severity levels of maintenance requirements. Results show that they can produce quality maintenance plans quickly, and RL proved to be effective in optimizing the maintenance decision process. Additionally, the fact that the two algorithms were designed for different horizon intervals indicates they can be used together and complementary to each other.

Aircraft maintenance practices can be continuously improved, and the benefits for the airlines of optimizing maintenance planning should encourage further research. Different approaches, e.g., meta-heuristic algorithms, could be pursued. Future work may also include the use of maintenance variables that were not considered in this paper: hangar availability, workforce availability and specialization, and readiness of maintenance parts and tools.

## Data Availability

The dataset was made available as part of the REMAP project mentioned in the acknowledgments (https://h2020-remap.eu), inquiries can be done using the contact form in the project’s webpage or the corresponding author.
